# Embryonic thermal manipulation improved early immunity in broiler chickens

**DOI:** 10.3389/fphys.2025.1670073

**Published:** 2025-09-17

**Authors:** Sadid Al Amaz, Suman Poudel, Bal Krishna Pandey, Suresh Burlakoti, Rajesh Jha, Birendra Mishra

**Affiliations:** Department of Human Nutrition, Food and Animal Sciences, College of Tropical Agriculture and Human Resilience, University of Hawaiʻi at Manoa, Honolulu, HL, United States

**Keywords:** cytokines, embryogenesis, immunomodulation, lymphoid, ontogeny

## Abstract

Immunity in chickens is age-dependent and develops gradually over time. The initial defense by the host is a crucial mechanism for combating microbial infections. Embryonic thermal manipulation (TM) represents a promising approach for sustainable broiler production. Our previous work provided significant insights into the effects of TM on embryonic thermotolerance, metabolism, growth performance, microbial diversity, and immunity. This follow-up study used a subset of hatchlings previously used in our TM-related studies. This study aims to investigate the effects of TM on immunity-related genes in the spleen, bursa, and thymus. A total of 600 fertile Cobb 500 eggs were incubated for 21 days. After candling, 238 eggs underwent TM at 38.5 °C from embryonic day (ED) 12–18, then transferred to a hatcher at 37.5 °C from ED 19 to 21, while 236 eggs were incubated at 37.5 °C throughout till 21 days. After hatching, 60-day-old unsexed chicks were housed in 12 pens (10 birds/pen, 6 replicates per treatment). The treatments included 1) Control and 2) TM. All birds were raised under standard conditions for the first 21 days. In the spleen, at d 7, the TM group showed significantly lower expression of cytokines (*IL-10, IL-12, IL-18), TLRs* (*TLR-1, TLR-2A, TLR-4, TLR-21*), and signaling markers (*TBK-1, CD-3, NF-κB, TGF-β, TGF-β3*) compared to the Control. At d 21, TM birds exhibited significantly lower (*P* < 0.05) expression of *IL-4, IL-6, IFN-γ*, and *AvBD-6*, while *TLR-2A* and *TGF-β3* were significantly upregulated compared to the Control group. In bursa, at d 7, the TM group showed significantly higher expression of *IL-1β, TLR-5, TLR-15, TLR-21, IFN-α,* and *NF-κB*, while *IL-6* was significantly downregulated. At d 14, *IL-18* was significantly upregulated, and *TLR-21* was significantly downregulated in the TM group. At d 21, *IL8L1, IL-10, TLR-1,* and *CD-45* were significantly upregulated, whereas *NF-κB* expression was significantly downregulated compared to the Control group. In the thymus, at d 14, *TLR15* was significantly higher, and at d 21, *IL-10* was significantly lower in the TM compared to the Control group. In conclusion, embryonic TM enhanced early immune gene expression in broilers by upregulating essential immune-related genes in the spleen, bursa, and thymus.

## 1 Introduction

Avian immune organs are classified into core immune organs, which include the thymus, bursa of Fabricius, bone marrow, and peripheral immune organs, such as the spleen and cecal tonsils. The maturation of the immune system in broiler chickens is age-dependent. In the early stages, the immune system and its functions are underdeveloped and do not mature until later stages (Song et al., 2021). The development and evolution of immune organs significantly affect the overall immune function of birds and their ability to withstand various antigens and environmental stresses (Naukkarinen and Hippeläinen, 1989).

The avian spleen is the primary location for lymphocyte differentiation and proliferation, actively participating in hormonal and cell-mediated responses. Furthermore, the avian spleen activates innate and adaptive immune responses, highlighting its significance in immune regulation (Smith and Hunt, 2004). The bursa serves as the center for B-cell lymphopoiesis, lymphocyte maturation, and the differentiation and maturation of the antibody. It acts as a primary channel by which environmental antigens activate the immune system and promote the production of specific antibodies (Ifrah et al., 2017). As the initial lymphoid organ emerges during organismal development, the thymus houses essential immunological components pertinent to innate and adaptive immune responses (Reese et al., 2006).

The embryonic thermal manipulation (TM) has been shown to improve heat tolerance in poultry (Al Amaz and Mishra, 2024). Subjecting embryos to elevated incubation temperatures during critical developmental phases can improve their immune response at a later stage (Al-Zghoul et al., 2023). Our previous studies have revealed that embryonic TM enhances hatchability, thermotolerance, and liver metabolism while reducing hatch duration (Amaz et al., 2024). In slow-growing chickens, genetics and environmental factors interact to influence adaptability to thermotolerance (Nayak et al., 2024). Additionally, prehatch TM and post-hatch baicalein supplementation enhanced final growth performance, cecal microbial diversity, volatile fatty acid concentration (Al Amaz et al., 2024a), liver metabolism, muscle cell proliferation (Al Amaz et al., 2024b), and immunity at the later stage (Al Amaz et al., 2025) in heat-stressed broilers. Given the effectiveness of embryonic TM, we hypothesized that it would improve the early immune response. This study investigated the effect of TM on early immune response in the spleen, bursa, and thymus by assessing the crucial immunity-related gene markers.

## 2 Materials and methods

### 2.1 Incubation

All experimental procedures involving animals were performed following the guidelines and regulations approved by the Institutional Animal Care and Use Committee (IACUC) of the University of Hawaiʻi (Approval No. 17-2605-6). This research utilized animal experimentation and samples from our previous studies (Amaz et al., 2024). Briefly, six hundred fertilized Cobb 500 broiler eggs were procured from Asagi Hatchery Inc (Honolulu, HI). The eggs were randomly and equally allocated among three incubators (GQF Incubator, Savannah, GA), each containing 200 eggs, despite the total capacity of the incubators being 270 eggs. The eggs were incubated under standard conditions (37.5 °C, 55% relative humidity (RH), 24 h/d) until embryonic day (ED) 11. Following candling, 474 viable embryos were chosen for the experiment. On ED 12, the eggs were allocated into two incubation cohorts: the Control group (n = 236), which maintained standard temperature until hatching on ED 21, and the TM group (n = 238), which underwent exposure to an elevated temperature at 38.5 °C and 55% relative humidity (RH) for 8 h/days from ED 12 to ED 18, subsequently reverting to standard temperature (37.5 °C) from ED 19 to ED 21. During ED 12-18, we employed only two incubators: one designated for the control group and another for the TM group. The incubation procedure involved automated temperature control, 55% RH, and egg rotation every 2 h.

### 2.2 Hatching and rearing management

The hatching rate was 91% in the Control and 94.5% in the TM cohorts (Amaz et al., 2024). After hatching, unsexed day-old chicks from both groups were divided into two treatment groups: 1) Control and 2) TM. The chicks were individually weighed, tagged, and randomly distributed into 12 pens, with 10 birds per pen, resulting in 6 replicates for each treatment group (n = 60 birds per treatment). Standard Cobb-500 broiler rearing and management protocols were followed (Cobb Broiler Management Guide, 2021). The Control and TM groups were maintained at a consistent 22 °C–24 °C temperature with 55% RH throughout the study. Birds were monitored thrice daily (morning, afternoon, and evening) to ensure health and management standards (discomfort, panting, or unusual behavior). Pens were randomly assigned, each 1 m × 0.61 m, providing a stocking density of 610 cm^2^ per bird. Wood shavings were used as litter (depth 4 in), the feeder’s dimensions were 15.75″L × 5.12″W × 4″H, and a hanging tube-4-nipple system (length 27″) waterer was used with the hose to a five-gallon bucket. The waterers were hung from the broader side of the pen and adjusted regularly, in accordance with the standard Cobb broiler protocol. There was no significant difference in growth performance during the brooding period. The birds were raised under a 23-h light, 1-h dark lighting schedule.

### 2.3 Diet

The corn-soybean meal-based basal diets were made for the starter phase (d 1–21) to fulfill the nutritional needs of the Cobb 500 broilers (Cobb Broiler Management Guide, 2021). Feed and water were provided *ad libitum* throughout the study. All the groups administered the basal diet for the duration of the study. The composition and nutrient profile of the diets are displayed in Table 1.

**TABLE 1:** Composition of experimental diets and their nutrient profile.

Ingredients, %	Starter diet (1–21 days)
	Control and TM
Corn	53.67
SBM	38.00
Soybean oil	5.00
Limestone	1.35
Monocalcium phosphate	0.75
Lysine	0.18
Met	0.18
Thr	0.04
Tryptophan	0.00
Choline Cl	0.00
Nacl	0.20
Sodium bicarbonate	0.12
Vitamin + mineral mix^a^	0.50
Phytase	0.01
Total	100.00
Nutrient Contents in the diet %	
AMEn, kcal/kg	3,040
CP	21.47
Ca	0.91
Total P	0.71
AvP	0.45
Lys	1.32
Met	0.52
Cys	0.42
Thr	0.87
Trp	0.31
Met + Cys	0.92
Arg	1.55
Val	1.18
Ile	0.90
Leu	1.82
NDF	8.86
CF	3.84
Na	0.16
Cl	0.16
Choline (mg/kg)	1,371
dig Lys%	1.17
dig Met%	0.48
dig Thr%	0.67

^a^ Provides following nutrients (per kg of diet): vitamin A (trans-retinyl acetate), 10,000 IU; vitamin D_3_ (cholecalciferol), 3,000 IU; vitamin E (all-*rac*-tocopherol-acetate), 30 mg; vitamin B_1_, 2 mg; vitamin B_2_, 8 mg; vitamin B_6_, 4 mg; vitamin B_12_ (cyanocobalamin), 0.025 mg; vitamin K_3_ (bisulfate menadione complex), 3 mg; choline (choline chloride), 250 mg; nicotinic acid, 60 mg; pantothenic acid (D-calcium pantothenate), 15 mg; folic acid, 1.5 mg; betaíne anhydrous, 80 mg; D-biotin, 0.15 mg; zinc (ZnO), 80 mg; manganese (MnO), 70 mg iron (FeCO_3_), 60 mg; copper (CuSO_4_·5H_2_O), 8 mg; iodine (KI), 2 mg; selenium (Na_2_SeO_3_), 0.2 m.

### 2.4 Sample collection

On days 7, 14, and 21, 2 hours after feeding, one bird from each pen (n = 6 per treatment) was euthanized using carbon dioxide asphyxiation for tissue collection. Spleen, bursa, and thymus tissues were collected, immediately snap-frozen, and stored at −80 °C until RNA extraction.

### 2.5 Quantitative real-time PCR (qPCR)

The total RNA was isolated from the spleen, bursa, and thymus tissues. The RNA concentration was measured with a NanoDrop™ spectrophotometer (ThermoFisher Scientific, Madison, WI). Subsequently, transcribed into cDNA and analyzed through qPCR by the established protocol (Amaz et al., 2025). The primer sequences utilized for gene expression analysis are presented in Supplementary Table S1. The NCBI Primer-Blast tool designed gene-specific primers for expression analysis. A High-Capacity cDNA Reverse Transcription Kit (Applied Biosystems, Foster City, CA) was used to reverse-transcribe 1 μg of total RNA (20 μL reaction of RT mixture) into complementary DNA (cDNA), which was subsequently diluted with nuclease-free water (1:25). qPCR was carried out using the PowerUp SYBR Green Master Mix (Applied Biosystems, Foster City, CA, United States) and real-time PCR equipment (Applied Biosystems). To achieve a final reaction volume of 10 μL, the qPCR reaction mixture included 3 μL of cDNA, 5 μL of PowerUp SYBR Green Master Mix, and 1 μL of each forward and reverse primer at a concentration of 5 μmol. The qPCR reaction was conducted using the standard cycling mode. A melting curve analysis was conducted to validate the SYBR Green-based amplicon. Furthermore, the specificity of each primer pair was evaluated through 1% gel electrophoresis of the qPCR products. The analysis was performed in triplicate for three housekeeping genes: glyceraldehyde 3-phosphate dehydrogenase (GAPDH), beta-actin (β-actin), and TATA-box binding protein (TBP). The *TBP* expression was consistently stable across the CAM tissues. Post-amplification, the cycle threshold (Ct) values were documented, and gene expression levels were determined utilizing *TBP* as the reference gene, following the 2^−ΔΔCT^ method.

### 2.6 Statistical analysis

Gene expression was assessed using GraphPad (GraphPad Software, San Diego, CA). Following a two-way analysis of variance (ANOVA), the Tukey-HSD test was utilized to compare the means of various treatment groups. All data are presented as mean ± SEM. The criterion for statistical significance was set at *P* < 0.05.

## 3 Results

### 3.1 Spleen gene expression

**FIGURE 1:**
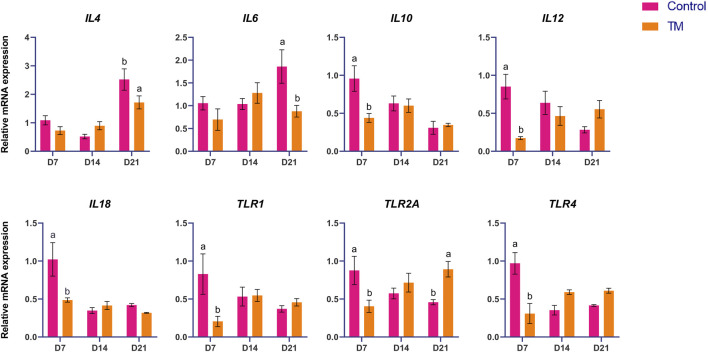
Effects of TM on the mRNA expression of immune-related genes in the spleen. Data are shown as mean ± SEM. Different letters indicate a significant difference between the treatment groups.

**FIGURE 2:**
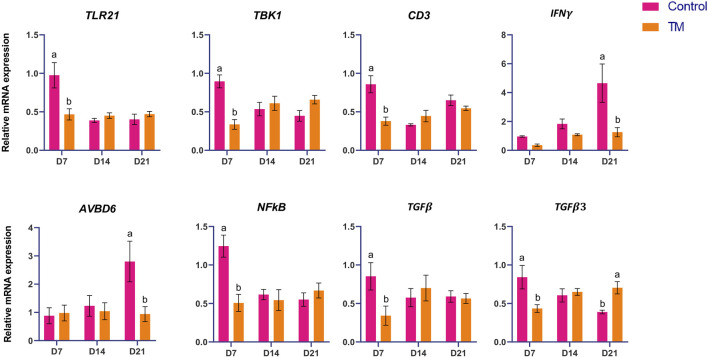
Effects of TM on the mRNA expression of immune-related genes in the spleen. Data are shown as mean ± SEM. Different letters indicate a significant difference between the treatment groups.

The expressions of immune-related genes (*IL-4, IL-6, IL-10, IL-12, IL-18, TLR-1, TLR-2A, TLR-4, TLR-21, TBK-1, CD-3, IFN-γ, AvBD-6, NF-kB, TGF-β,* and *TGF-β3*) among the treatments are shown in Figures 1, 2. At d 7, *IL-10, IL-12, IL-18, TLR-1, TLR-2A, TLR-4, TLR-21, TBK-1, CD-3, NF-kB, TGF-β*, and *TGF-β3* expressions were significantly lower (*P* < 0.05) in the TM group than the Control group. There was no significant difference at d 14. However, at d 21, *IL-4, IL-6, IFN-γ*, and *AvBD-6* expression were significantly lower (*P* < 0.05), and *TLR-2A* and *TGF-β3* expression were significantly higher (*P* < 0.05) in the TM group compared to the Control.

### 3.2 Bursa gene expression

**FIGURE 3:**
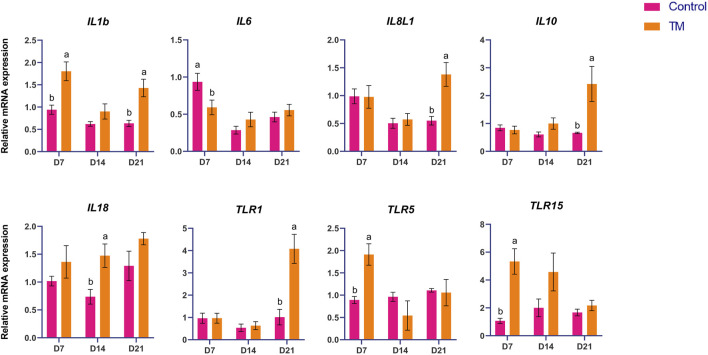
Effects of TM on the mRNA expression of immune-related genes in the bursa. Data are shown as mean ± SEM. Different letters indicate a significant difference between the treatment groups.

**FIGURE 4:**
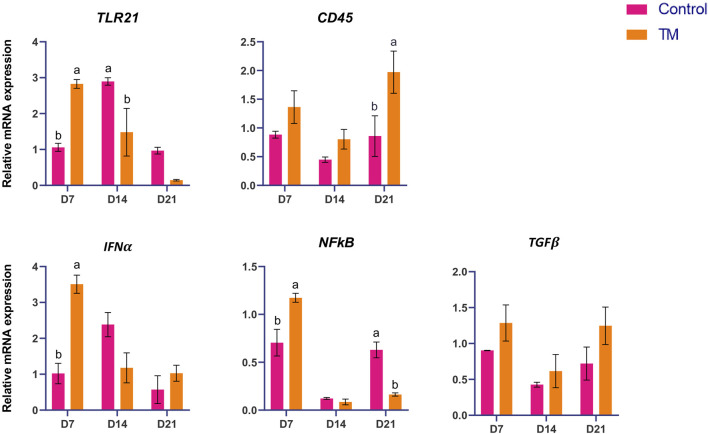
Effects of TM on the mRNA expression of immune-related genes in the bursa. Data are shown as mean ± SEM. Different letters indicate a significant difference between the treatment groups.

The expressions of immune-related genes (*IL-1b, IL-6, IL-8L1, IL-10, IL-18, TLR-1, TLR-5, TLR-15, TLR-21, CD-45, IFN-α, NF-kB,* and *TGF-β*) among the treatments are shown in Figures 3, 4. At d 7, *IL-1 b, TLR-5, TLR-15, TLR-21, IFN-α,* and *NF-kB* were significantly higher (*P* < 0.05) and *IL-6* was significantly lower (*P* < 0.05) in the TM group than the Control group. At d 14, *IL-18* was significantly higher (*P* < 0.05), and *TLR-21* was significantly lower (*P* < 0.05) in the TM group than in the Control group. At d 21, *IL8L1, IL-10, TLR-1*, and *CD-45* were significantly higher (*P* < 0.05); however, *NF-kB* was significantly lower (*P* < 0.05) in the TM group than in the Control group.

### 3.3 Thymus gene expression

**FIGURE 5:**
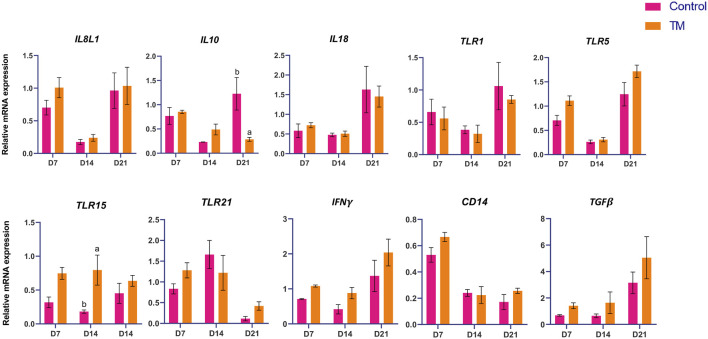
Effects of TM on the mRNA expression of immune-related genes in the thymus. Data are shown as mean ± SEM. Different letters indicate a significant difference between the treatment groups.

The expressions of immune-related genes (*IL-1b, IL-6, IL-8L1, IL-10, IL-18, TLR-1, TLR-5, TLR-15, TLR-21, CD-45, IFN-a, NF-kB,* and *TGF- β*) among the treatments are shown in Figure 5. At d 14, *TLR-15* was significantly higher (*P* < 0.05), and at d 21, *IL-10* was significantly lower (*P* < 0.05). There was no significant difference between the groups at d 7.

## 4 Discussion

The largest peripheral lymphoid organ in chickens is the spleen, which plays a crucial role in immune responses, particularly to blood-borne antigens (Kraal, 1992). In this study, we measured the expression of essential immune-related genes (*IL-4, IL-6, IL-10, IL-12, IL-18, TLR-1, TLR-2A, TLR-4, TLR-21, TBK-1, CD-3, IFN-γ, AvBD-6, NF-κB, TGF-β,* and *TGF-Β3*) in the spleen. *IL-10* may be pivotal in altering the Th bias during E. maxima infection, inhibiting the emergence of robust, IFN-γ-driven responses, which are essential for managing Eimeria infections (Rothwell et al., 2004). In this study, at d 7, *IL-10* expression was significantly reduced in TM birds compared to Controls, meaning the TM group may promote a more protective, pro-inflammatory environment, potentially shifting the local immune response from a Th2-biased, anti-inflammatory profile at an early stage. The *IL-12* in chickens is essential for the immune response, especially in the spleen, by facilitating Th1-type immunity and the synthesis of IFN-γ. It plays a role in activating and proliferating specific T cell populations, such as γδ T cells. It is expressed in the spleen, particularly during infections such as coccidia (Degen et al., 2004). *IL-12* expression was significantly lower in the TM than in the Control group at d 7. Its suppression could indicate a delayed development of early cell-mediated immunity. *IL-18* interacts with *IL-12* to stimulate IFN-γ production from T-helper 1 (Th1) cells and natural killer cells (Tominaga et al., 2000). *TLR-1* is crucial for the identification of triacyl lipopeptides. Mice deficient in TLR-1 macrophages demonstrated diminished synthesis of inflammatory cytokines upon exposure to diverse triacyl lipopeptides and lipoproteins originating from mycobacteria (Kannaki et al., 2010). The function of TLR-4 as the receptor for gram-negative lipopolysaccharide is well recognized. Moreover, it binds to endogenous molecules produced due to tissue injury. Consequently, TLR-4 is an essential receptor that integrates infectious and noninfectious stimuli to initiate an inflammatory response (Molteni et al., 2016). TLR-21 is a pattern recognition receptor that identifies microbial DNA to initiate the host’s immune response to infection (Chuang et al., 2020). In chicken, chTBK1 might be a crucial immunoregulator for the induction of IRF-3 and IFN-β in response to virus stimulation (Wang et al., 2017). *NF-kB* is the principal regulator of the innate immune response to pathogenic bacteria, activated through MyD88-dependent signaling within the TLR pathway (Moynagh, 2005). TGF-βs regulate pro-inflammatory and anti-inflammatory activities, demonstrating their dual roles in the immune system (Okamura et al., 2015). At d 7, *IL-18, TLR-1, TLR-4, TLR-21, TBK-1, CD-3. NF-kB* and *TGF-β* were significantly downregulated in the TM compared to the control group. This coordinated downregulation indicates a reduced capacity for pathogen recognition and signaling, possibly due to the attenuation of MyD88-and IRF3-dependent pathways. The lower expression of *IL-18* and *IL-12* may impede IFN-γ production, consequently restricting Th1 and NK cell activation. These findings suggest that TM induces an immunomodulatory state after hatching, likely to reduce early-life inflammation and energy expenditure in the absence of infection, while altering the trajectory of immune system maturation. *IL-4* is a cytokine that regulates antibody synthesis, hematopoiesis, inflammation, and the development of effector T-cell responses (Brown and Hural, 1997). *IL-6* facilitates a transient defense against infection or injury by alerting the immune system to the source of inflammation. It modulates the immune response by facilitating the proliferation and differentiation of leukocytes that eradicate pathogenic microorganisms (Rodes, 2013). *IFN-γ* activates macrophages to augment phagocytosis, tumoricidal functions, and intracellular eradication of pathogens. It prompts macrophages to generate a diverse array of inflammatory mediators and reactive oxygen and nitrogen species. (IFN-*γ*) amplifies the cellular immune response after infection and vaccination (Santhakumar et al., 2017; Bagheri et al., 2022). In this study, at d 21, *IL-4, IL-6, TLR-2A, IFN-γ, and AvBD-6* were significantly downregulated, and *TGF-β3* was significantly upregulated in the TM compared to the control group. This expression profile signifies a transition towards a regulatory or anti-inflammatory immune condition. The inhibition of *IL-4* and *IL-6* indicates diminished B-cell activation and inflammatory signaling, whereas lowered *IFN-γ* and *AvBD-6* levels suggest reduced macrophage activation and antimicrobial peptide defense. The simultaneous increase of *TGF-β3*, an essential immunosuppressive cytokine, facilitates the development of immune tolerance and the resolution of inflammation. Taken together, TM modified the course of immune development by attenuating early inflammatory signaling and immune activation in the spleen. On d 7, TM reduces innate immune responsiveness, whereas by d 21, suggesting a sustained shift toward immune homeostasis during early development. This immune modulation persisted until the marketing age (d 35) (Al Amaz et al., 2025). It proved advantageous by preserving energy for growth (Al Amaz et al., 2024a), as observed in our previous study with the same set of birds.

This study assessed the expression of crucial immune-related genes (*IL-1β, IL-6, IL-8L1, IL-10, IL-18, TLR-1, TLR-5, TLR-15, TLR-21, CD-45, IFN-α, NF-κB,* and *TGF-β*) in the bursa. *IL-1b* is an essential mediator of inflammation. In addition to being essential for the host’s defense against infections, it intensifies damage during acute tissue injury and chronic diseases (Lopez-Castejon and Brough, 2011). *IL-1b* was significantly upregulated in the TM compared to the control group on days 7 and 21, indicating an activated pro-inflammatory condition that may augment initial immune surveillance and pathogen defense. *TLR-5* is essential for recognizing bacterial flagellin and triggering pro-inflammatory signaling via NF-κB activation. Its expression in diverse chicken tissues and immune cells, including heterophils and macrophage-like HD11 cells, enhances cytokine and chemokine production in response to bacterial exposure (Rehman et al., 2021). *TLR-15* and -*21* may be linked to the resistance or susceptibility of chickens to bacterial infections, including *Salmonella* (Ruan et al., 2012). At d 7, *TLR-5, TLR-15,* and *IFN-α* were significantly upregulated, while *TLR-21* was significantly upregulated at d 7 and significantly downregulated at d 14. Indicating the early activation of innate immune detection and antiviral mechanisms. *NF-kB* also followed the same pattern. It was significantly upregulated at d 7 and significantly downregulated at d 21 in the TM. *TLR21* and *NF-kB* are probably upregulated on d 7 to improve early microbial recognition and immune preparedness, suggesting a temporary activation of pro-inflammatory signaling and subsequently downregulated by days 14 and 21 as a feedback mechanism to sustain immune equilibrium and avert overstimulation once initial microbial exposure has been mitigated. In chickens, *IL-8L1* is part of the CXC family and primarily influences neutrophils, T cells, B cells, and other lymphocytes (Van Sweringen et al., 2011). *CD-45* regulates the interaction between T cells and macrophages by engaging with the ligand, macrophage galactose-type lectin. This interaction involves binding to CD-45 N-acetyl galactosamine, leading to diminished T cell proliferation and heightened pro-inflammatory cytokine production, ultimately culminating in T cell apoptosis (Rožman and Švajger, 2018). At d 21, *IL-8L1, IL-10, TLR-1,* and *CD-45* were significantly upregulated in the TM group compared to the control group. This pattern indicates a transition to a more actively immunologically regulated condition, potentially preserving tissue homeostasis while facilitating pathogen defense in later development (Al Amaz et al., 2025). TM elicited a dynamic immune response in the bursa, marked by the early activation of innate defense and microbial-sensing pathways within the first week post-hatch. This preliminary response likely improves immune preparedness as chicks confront environmental antigens. In the later stages, the immune profile transitioned to a more regulated and balanced condition, facilitating immune surveillance and tissue homeostasis.

In the thymus, the expressions of immune-related genes (*IL-1b, IL-6, IL-8L1, IL-10, IL-18, TLR-1, TLR-5, TLR-15, TLR-21, CD-45, IFN-α, NF-kB,* and *TGF-β*) were assessed. *TLR-15* was significantly upregulated at d 14, and *IL-10* was significantly downregulated at d 21 in the TM compared to the Control group. In the thymus, TM resulted in a delayed upregulation of innate immune sensing on d 14, succeeded by reduced anti-inflammatory signaling on d 21. This pattern indicates a transition towards increased immune activation and diminished immune regulation, likely facilitating more robust T-cell maturation and responsiveness in later development.

## 5 Conclusion

This study revealed that embryonic thermal manipulation (TM) induces organ- and time-specific modulation in early immune development in chickens. In the spleen, TM suppressed pro-inflammatory and pathogen recognition signals, signifying the inhibition of MyD88-and IRF3-dependent pathways. TM increased innate immune detection and cytokine synthesis in the bursa, indicating the preliminary activation of antimicrobial defenses. Thymus responses exhibited lower regulatory signaling and higher innate activation, which may promote T-cell maturation. TM appeared to enhance immune function by fostering early immune response while establishing long-term immune balance, which may contribute to improved disease resistance and growth efficiency in later life stages.

## Data Availability

The datasets presented in this study can be found in online repositories. The names of the repository/repositories and accession number(s) can be found in the article/Supplementary Material.
