# Supplementary material for: Embryonic thermal manipulation improved early immunity in broiler chickens
Source: Front Physiol. 2025 Sep 17;16:1670073. doi: 10.3389/fphys.2025.1670073 (PMC12484176; doi:10.3389/fphys.2025.1670073)
Supplement: Supplementary file 1 [file Table1.docx]

**Supplement Table 1:** List of primers used in qPCR.

| Gene | Accession No. | Primer sequence | Amplicon length |
| --- | --- | --- | --- |
| *TBP* | XM_025148547.3 | F: TAGCCCGATGATGCCGTAT | 147 |
|  |  | R: GTTCCCTGTGTCGCTTGC |  |
| *GAPDH* | NM_204305.2 | F: AGCTTACTGGAATGGCTTTCCG | 122 |
|  |  | R: ATCAGCAGCAGCCTTCACTACC |  |
| *B-Actin* | NM_205518.2 | F:GAG AAA TTG TGC GTG ACA TCA | 152 |
|  |  | R: CCT GAA CCT CTC ATT GCC A |  |
| *IL1b* | NM_205064.1 | F: TGCCTGCAGAAGAAGCCTCG | 204 |
|  |  | R: GACGGGCTCAAAAACCTCCT |  |
| *Il8L1* | NM_205018.2 | F: GCTGGAGCAAAAGGTATGGC | 214 |
|  |  | R: TGATCAGCTTCACCCAGGGA |  |
| *IL4* | NM_001030693 | F: TGTGCCCACGCTGTGCTTACA | 155 |
|  |  | R: CTTGTGGCAGTGCTGGCTCTCC |  |
| *IL6* | NM_204628.2 | F: TTCACCGTGTGCGAGAACAGC | 80 |
|  |  | R: CAGCCGTCCTCCTCCGTCAC |  |
| *IL10* | NM_001004414 | F: TGTCACCGCTTCTTCACCTG | 105 |
|  |  | R: CTCCCCCATGGCTTTGTAGA |  |
| *IL12* | NM_213571 | F:AGA CTC CAA TGG GCA AAT GA | 113 |
|  |  | R:CTC TTC GGC AAA TGG ACA GT |  |
| *IL18* | XM_046932263.1 | F: AGATGATGAGCTGGAATGCGATGC | 97 |
|  |  | R: ATCTGGACGAACCACAAGCAACTG |  |
| *TLR1* | [NM_001081709.4](https://www.ncbi.nlm.nih.gov/entrez/viewer.fcgi?db=nucleotide&id=2099395557) | F: AAGCTTCCCGTGGAGTGATG | 96 |
|  |  | R: GAAACCCAAGGTGGAGGAGG |  |
| *TLR2A* | NM_205064.1 | F: TGCCTGCAGAAGAAGCCTCG | 204 |
|  |  | R: GACGGGCTCAAAAACCTCCT |  |
| *TLR4* | [NM_001030693.2](https://www.ncbi.nlm.nih.gov/entrez/viewer.fcgi?db=nucleotide&id=2099396323) | F: CCAAACACCACCCTGGACTT | 227 |
|  |  | R: AGCGACGTTAAGCCATGGAA |  |
| *TLR5* | XM_046914372.1 | F: TCCATCCTGGAGGAGCGTTA | 320 |
|  |  | R: GTGCTCCAGCCATTCTCACT |  |
| *TLR15* | NM_001398239.1 | F: CTCGGGGCCTTCACAGATTT | 344 |
|  |  | R: GATCCATCTCCAGGTCGCTG |  |
| *CD14* | NM_001139478.2 | F: TGGACGACTCCACCATTGAC | 132 |
|  |  | R: CCATCTCCTGCACCTGAGTG |  |
| *TLR21* | XM_040707023.2 | F: ACCTGCTGACCGACCTCTAT | 466 |
|  |  | R: AGCATGTTCTGTGACAGCGA |  |
| *CD3* | NM_205512 | F: GGACGCTCCCACCATATCAG | 180 |
|  |  | R: TGTCCATCATTCCGCTCACC |  |
| *CD45* | NM_204417 | F: TATTCTTGGTGTTCTTGATTGTTGTG | 120 |
|  |  | R: CTGCTACAAGGCTGATGACTTCA |  |
| *TBK1* | NM_001139478.2 | F: TGGACGACTCCACCATTGAC | 132 |
|  |  | R: CCATCTCCTGCACCTGAGTG |  |
| *TGFb* | NM_204628.2 | F: TTCACCGTGTGCGAGAACAGC | 80 |
|  |  | R: CAGCCGTCCTCCTCCGTCAC |  |
| *TGFb3* | [NM_001081709.4](https://www.ncbi.nlm.nih.gov/entrez/viewer.fcgi?db=nucleotide&id=2099395557) | F: AAGCTTCCCGTGGAGTGATG | 96 |
|  |  | R: GAAACCCAAGGTGGAGGAGG |  |
| *AvBD6* | NM_001004414 | F: TGTCACCGCTTCTTCACCTG | 105 |
|  |  | R: CTCCCCCATGGCTTTGTAGA |  |
| *NFkB* | NM_001396038.1 | F:GTGTGAAGAAACGGGAACTG | 203 |
|  |  | R:GGCACGGTTGTCATAGATGG |  |
| *IFNa* | XM_046936225.1 | F: GATGGCCTCGCAACCTTCA | 330 |
|  |  | R: AGGGGCTTCTGTGATTGAGC |  |
| *IFNg* | [NM_001030693.2](https://www.ncbi.nlm.nih.gov/entrez/viewer.fcgi?db=nucleotide&id=2099396323) | F: CCAAACACCACCCTGGACTT | 227 |
|  |  | R: AGCGACGTTAAGCCATGGAA |  |

F=Forward primer, R=Reverse primer
